# Loiasis in sub-Saharan migrants living in Spain with emphasis of cases from Equatorial Guinea

**DOI:** 10.1186/s40249-020-0627-4

**Published:** 2020-02-07

**Authors:** Sabino Puente, German Ramírez-Olivencia, Mar Lago, Mercedes Subirats, Francisco Bru, Eugenio Pérez-Blazquez, Marta Arsuaga, Concepción Ladron de Guevara, Fernando de la Calle-Prieto, Belén Vicente, Montserrat Alonso-Sardón, Moncef Belhassen-Garcia, Antonio Muro

**Affiliations:** 1 Unidad de Medicina Tropical. Servicio de Medicina Interna. Hospital La Paz-Carlos III, Madrid, Spain; 2 Unidad de Aislamiento de Alto Nivel. Sección de Enfermedades Infecciosas. Servicio de Medicina Interna. Hospital Central de la Defensa Gómez Ulla, Madrid, Spain; 3 Microbiología. Hospital La Paz-Carlos III, Madrid, Spain; 4 Dermatología, Madrid, Spain; 5 Servicio de Oftalmología. Hospital Universitario 12 de Octubre, Universidad Complutense de Madrid, Madrid, Spain; 6 Laboratorio de Inmunología Parasitaria y Molecular. CIETUS. IBSAL, Facultad de Farmacia, Universidad de Salamanca, Avenida Donantes de Sangre s/n, 37007 Salamanca, Spain; 7 Área de Medicina Preventiva y Salud Pública, IBSAL, CIETUS, Universidad de Salamanca, Salamanca, Spain; 8 Servicio de Medicina Interna. Sección de Enfermedades Infecciosas. CAUSA. IBSAL. CIETUS, Universidad de Salamanca, Paseo San Vicente 58-182, 37007 Salamanca, Spain

**Keywords:** *Loa loa*, Loiasis, Tropical medicine, Clinical study, Immigrant, Imported disease, Spain

## Abstract

**Background:**

Loiasis is an uncommon and poorly understood parasitic disease outside endemic areas of Africa. The aim of this study was to describe the clinical and biological patterns and treatment of imported loiasis by sub-Saharan migrants diagnosed in Madrid, Spain.

**Methods:**

A retrospective study was conducted with sub-Saharan immigrants seen at the Tropical Medicine Unit of the Carlos III Hospital in Madrid, Spain, a reference center, over 19 years. Categorical variables were expressed as frequency counts and percentages. Continuous variables were expressed as the mean and standard deviation (*SD*) or median and interquartile range (IQR: Q3–Q1). Chi-square tests were used to assess the association between categorical variables. The measured outcomes were expressed as the odds ratio (OR) with a 95% confidential interval. Continuous variables were compared by Student’s t-tests or Mann-Whitney U tests. Binary logistic regression models were used. *P* <  0.05 was considered a statistically significant difference.

**Results:**

One hundred thirty-one migrants from tropical and subtropical areas with loiasis were identified. Forty-nine patients were male (37.4%). The migrants’ mean age (±*SD*) was 42.3 ± 17.3 years, and 124 (94.7%) were from Equatorial Guinea. The median time (IQR) between arrival in Spain and the first consultation was 2 (1–7) months. One hundred fifteen migrants had eosinophilia, and one hundred thirteen had hyper-IgE syndrome. Fifty-seven patients had pruritus (43.5%), and thirty patients had Calabar swelling (22.9%). Seventy-three patients had coinfections with other filarial nematodes (54.2%), and 58 migrants had only *Loa loa* infections (45.8%). One hundred two patients (77.9%) were treated; 45.1% (46/102) patients were treated with one drug, and 54.9% (56/102) patients were treated with combined therapy. Adverse reactions were described in 14 (10.7%) migrants.

**Conclusions:**

Our patients presented early clinical manifestations and few atypical features. Thus, physicians should systematically consider loiasis in migrants with a typical presentation. However, considering that 72.5% of the patients had only positive microfilaremia without any symptoms, we suggest searching for microfilaremia in every migrant from endemic countries for loiasis presenting with eosinophilia.

## Introduction

Loiasis, a filarial infection caused by *Loa loa,* is transmitted by the bite of adult female *Chrysops* flies. Loiasis, also called the “*African eye worm*”, affects between 3 and 13 million individuals living in west and central regions of Africa [1, 2]. The endemic countries are Angola, Chad, Democratic Republic of the Congo, Cameroon, Central African Republic, Equatorial Guinea, Gabon, Nigeria, Republic of Congo, and South Sudan [3]. In endemic areas, loiasis is often regarded as benign, and the infection is so common that little effort has been made to assess the frequency of its clinical manifestations and the efficacy of the various treatments used [4, 5]. Nevertheless, Chesnais and colleagues [6] showed an excess of mortality associated with loiasis. Imported filariasis is an uncommon and poorly known parasitic disease in developed countries. Therefore, the Geosentinel network identified 271 (0.62%) cases of filariasis among 43 772 imported diseases from 1995 to 2004 [7], with loiasis representing 25% of the cases [7]. The clinical symptoms are transient and mild in the majority of symptomatic patients [8, 9]. There are two classical profiles: i) ocular or subcutaneous crossing of an adult worm, which is the main clinical finding and resolves spontaneously; and ii) Calabar swelling, which is transient, localized angioedema due to hypersensitivity reactions to migrating adult or microfilariae. Other described complications are encephalitis, cardiomyopathy, nephropathy, arthritis, and lymphadenitis. Loiasis occurs most commonly in residents of endemic areas, but tourists and expatriates who live more than 6 months in endemic countries can be infected, although this is uncommon. A clinical spectrum of loiasis cases has been mainly described in case reports. They showed that the infection features differ markedly between endemic areas and travelers. Nonimmune individuals who travel to endemic regions and acquire *L. loa* infections are more prone to allergic-type symptoms than local residents [8].

The aim of this study was to describe the clinical and biological patterns and treatment of imported loiasis by sub-Saharan migrants diagnosed at the Tropical Medicine Unit of the Carlos III Hospital in Madrid, Spain.

## Methods

### Study design

The La Paz-Carlos III Hospital in Madrid, Spain, is a tropical disease referral unit. Most patients voluntarily go the emergency unit or are referred from primary care or general hospitals in Madrid. The center is visited by 300 migrants per year, and 80% of them are from Equatorial Guinea. A total of 5700 migrants visited over a 19-year period. A very small percentage of patients come from other regions.

A retrospective study was conducted on the data regarding immigrants diagnosed with loiasis over a 19-year period. The data included demographics (age, sex, nationality, time of the first consultation) and clinical characteristics (symptoms and when the symptoms first appeared). The eye examination results and analytical data regarding commercial serologic tests for syphilis, HIV, hepatitis B and C, eosinophil count, IgE levels and stool tests (Kato-Katz) regarding ova and parasites were reviewed. Other laboratory test results were also recorded.

Systematic ophthalmology exploration was performed in patients with a clinical suspicion of onchocerciasis. Relative eosinophilia was defined as an elevated percentage of eosinophils (> 5%) in individuals with < 450 × 10^6^ eosinophils/L. Absolute eosinophilia was defined as an increase in the peripheral blood eosinophilic leukocytes to more than 450 × 10^6^ eosinophils/L of blood. Mild eosinophilia was defined as (450 × 10^6^–999 × 10^6^ eosinophils/L). Moderate eosinophilia was defined as (1000 × 10^6^–2999 × 10^6^ eosinophils/L), and high eosinophilia was defined as > 3000 × 10^6^ eosinophils/L. Hyper-IgE syndrome was defined as an increase in peripheral blood IgE to more than 200 U/ml. Hyper-IgE syndrome was classified as mild (> 200–399 U/ml), moderate (> 399–999 U/ml) and/or high (> 1000 U/ml).

The diagnosis of loiasis was established with the presence of suggestive clinical manifestations (worm ocular migration, Calabar swellings) and/or confirmed microfilaremia or identification of adult worms following extraction. The direct detection of circulating microfilaria was performed on fresh venous blood obtained around midday with a thick film and/or thin smear after Giemsa stain; microfilaremia was occasionally estimated on thin smear [10].

The exclusion criteria were as follows: i) diagnosis in travelers, ii) unspecified diagnosis methods, and iii) medical records with missing data.

### Statistical analysis

Categorical variables were expressed as frequency counts and percentages. Continuous variables were expressed as the mean and standard deviation (*SD*) or median and interquartile range (IQR: Q_3_–Q_1_). Chi-square tests were used to assess the association between categorical variables (i.e., clinical and demographic variables). The measured outcomes were expressed as the odds ratio (*OR*) with a 95% confidential interval (*CI*). Continuous variables were compared by Student’s *t*-tests or Mann-Whitney U tests for two groups depending on if they were normally or non-normally distributed. Binary logistic regression models were used to associate binary outcome variables and the two exposure groups, and *P* <  0.05 was considered a statistically significant difference. The Statistical Package for the Social Sciences (SPSS 23.0®; IBM Corp., Armonk, New York, USA) was used to analyze all of the data.

## Results

### Epidemiological data

One hundred thirty-one cases of loiasis were identified. Forty-nine patients were male (37.4%), and 82 were females (62.6%). The patients’ mean age (±*SD*) was 42.3 ± 17.3 years [median (IQR), 41 (57–28) years]. The patient epidemiological data are shown in Table 1. Equatorial Guinea, Cameroon, Nigeria DR Congo, Gabon African RC were the countries of origin. The mean time (±*SD*) between the arrival of the patients to Spain and their first consultation was 10.9 ± 22.8 months [median (IQR): 2 (1–7) months].

**Table 1:** Main epidemiological data of patients included in the study

Demographic data	Migrants (*N* = 131)
Age, years	
Mean ± *SD*	42.3 ± 17.3
Median (IQR: Q_3_–Q_1_)	41 (57–28)
Range (Minimum value, Maximum value)	(16, 88)
< 45 years old	77 (58.8)
≥ 45 years old	54 (41.2)
Sex, *n* (%)	
Female	82 (62.6)
Male	49 (37.4)
Race, *n* (%)	
Black	130 (99.2)
Mix raze (black & white)	1 (0.8)
Origin country, *n* (%)	
Equatorial Guinea	123 (93.9)
Africa, other	8 (6.1)
Infection country, *n* (%)	
Equatorial Guinea	124 (94.7)
Africa, other	7 (5.3)
Time to first assessment in consultation, months	
Mean ± *SD*	10.9 ± 22.8
Median (IQR, Q_3_–Q_1_)	2 (7–1)
Range (Minimum value, Maximum value)	(1, 119)
Years of residence in endemic area, years	
Mean ± *SD* (range)	42.2 ± 17.3
Median (IQR: Q_3_–Q_1_)	41 (57–27)
Range (Minimum value, Maximum value)	(16, 88)

*SD* Standard deviation, *IQR* Interquartile range

### Clinical and laboratory data

Table 2 shows the main clinical and laboratory data of the patients. These data were stratified according to the risk markers sex and age. Regarding the clinical manifestations, 57 (43.5%) patients had pruritus, 30 (22.9%) had Calabar swelling observed by a clinician [upper extremities (20), face (7) and lower extremities (3)]; 19 (14.5%) had eye worms, 12 (9.2%) had arthralgia, 4 (3.1%) had abdominal pain, and 3 (2.3%) had subcutaneous nonpitting and nontender edema. When comparing the percentage of patients with symptomatic vs asymptomatic disease, no significant differences between men (59.2% vs 40.8%) and women (65.9% vs 34.1%) were found (*P* = 0.443), and no significant differences between an age <  45 years old (61.0% vs 39.0%) and ≥ 45 years old (66.7% vs 33.3%) were found (*P* = 0.511). However, some specific symptoms, such as Calabar swelling (30.5 vs 10.2, *P* = 0.008) and eye worms (20.7 vs 4.1, *P* = 0.009), were more frequent in women than in men. Other symptoms, such as pruritus (50.6 vs 33.3, *P* = 0.049), were more frequent in patients younger than 45 years old. Arthralgia (18.5 vs 2.6, *P* = 0.002) was common in patients over 45 years old.

**Table 2:** Main clinical, laboratory and diagnosis data of imported loiasis. Stratification of the data according to risk markers: sex and age

	Migrants (*N* = 131)	Sex			Age, years		
		Female (*n*_1_ = 82)	Male (*n*_2_ = 49)	*P*-value	< 45 (*n*_3_ = 77)	≥ 45 (*n*_4_ = 54)	*P*-value
Clinical data, *n* (%)							
12Asymptomatic	48 (36.6)	28 (34.1)	20 (40.8)	0.443	30 (39.0)	18 (33.3)	0.511
Symptomatic	83 (63.4)	54 (65.9)	29 (59.2)		47 (61.0)	36 (66.7)	
Calabar swelling	30 (22.9)	25 (30.5)	5 (10.2)	0.008*	14 (18.2)	16 (29.6)	0.125
Eye worm	19 (14.5)	17 (20.7)	2 (4.1)	0.009*	11 (14.3)	8 (14.8)	0.933
Subcutaneous step	3 (2.3)	2 (2.4)	1 (2.0)	0.883	2 (2.6)	1 (1.9)	0.779
Laboratory data, *n* (%)							
Eosinophilia (*n* = 131)							
Without eosinophilia	16 (12.2)	10 (12.2)	6 (12.2)	0.931	15 (19.5)	1 (1.9)	< 0.001*
Relative eosinophilia	4 (3.1)	3 (3.7)	1 (2.0)		3 (3.9)	1 (1.9)	
Mild eosinophilia	33 (25.2)	20 (24.4)	13 (26.5)		26 (33.8)	7 (13.0)	
Moderate eosinophilia	65 (49.6)	42 (51.2)	23 (46.9)		29 (37.7)	36 (66.7)	
High eosinophilia	13 (9.9)	7 (8.5)	6 (12.2)		4 (5.2)	9 (16.7)	
Immunoglobulin E (*n* = 129)							
Normal	16 (12.4)	12 (15.0)	4 (8.2)	0.394	11 (14.7)	5 (9.3)	0.056
Mild hyper-IgE	6 (4.7)	5 (6.3)	1 (2.0)		5 (6.7)	1 (1.9)	
Moderate hyper-IgE	29 (22.5)	16 (20.0)	13 (26.5)		21 (28.0)	8 (14.8)	
High hyper-IgE	78 (60.5)	47 (58.8)	31 (63.3)		38 (50.7)	40 (74.1)	
Direct diagnosis, *n* (%)							
Only Microfilariae	95 (72.5)	53 (64.6)	42 (85.7)	0.050	59 (76.6)	36 (66.7)	0.403
Microfilariae + Calabar swellings	7 (5.3)	3 (3.7)	4 (8.2)		2 (2.6)	5 (9.3)	
Microfilariae + eye	3 (2.3)	2 (2.4)	1 (2.0)		1 (1.3)	2 (3.7)	
Microfilariae + Calabar swellings + eye	5 (3.8)	5 (6.1)	0		4 (5.2)	1 (1.9)	
Microfilariae + eye + subcutaneous lesion	1 (0.8)	1 (1.2)	0		1 (1.3)	0	
Microfilariae + Calabar swellings + eye + subcutaneous lesion	1 (0.8)	1 (1.2)	0		0	1 (1.9)	
Only Calabar swellings	10 (7.6)	9 (11.0)	1 (2.0)		5 (6.5)	5 (9.3)	
Only eye	1 (0.8)	1 (1.2)	0		1 (1.3)	0	
Calabar swellings + eye	7 (5.3)	7 (8.5)	0		3 (3.9)	4 (7.4)	
Eye + subcutaneous lesion	1 (0.8)	0	1 (2.0)		1 (1.3)	0	

*P < 0.05

Overall, 87.8% (115/131) of the migrants had eosinophilia, and there was no difference between symptomatic (89.1%, 74/83 patients) and asymptomatic patients (85.4%, 41/48 patients). Regarding the levels of IgE, 86.2% (113/131) of migrants had hyper-IgE syndrome, and there was no difference between symptomatic (86.7%, 72/83 patients) and asymptomatic patients (85.4%, 41/48 patients).

Table 3 shows the coinfection of *Loa loa* with other parasites. Sixty migrants (45.8%) had only *Loa loa* infections; 71 patients (54.2%) had coinfections with other filarial nematodes. The presence of coinfections with other filarial nematodes was significantly related to sex (65.3% men vs 47.6% women, *P* = 0.049), but no significant differences (*P* > 0.05) were found between coinfections and age, clinical manifestations, eosinophilia and hyper-IgE syndrome.

**Table 3:** Co-infections in patients with loiasis

	Total, *N* (%)
Filarial co-infection*	
*Mansonella perstans*	50 (36.6)
*Onchocerca volvulus*	7 (5.3)
*Mansonella perstans + Onchocerca volvulus*	12 (9.2)
*Mansonella perstans + Onchocerca volvulus + Mansonella streptocerca*	3 (2.3)
*Mansonella perstans + Mansonella streptocerca + Wuchereria bancrofti*	1 (0.8)
Others helminthic co-infection	
Trichuris trichiura**	34 (26.0)
Ascaris lumbricoides**	20 (15.3)
*Hookworms*	11 (8.4)
Strongyloides stercoralis**	4 (3.1)
*Schistosoma* spp.***	0
Protozoa co-infection	
*Plasmodium* sp	22 (16.8)
Cyclospora cayetanensis**	1 (0.8)
Giardia lamblia**	0
Viruses co-infection	
HIV	16 (12.2)
HCV	10 (7.6)
HBV	4 (3.1)
Bacterial co-infection	
*Treponema pallidum*	4 (3.1)
*Mycobacterium* tuberculosis	3 (2.3)
*Mycobacterium leprae*	1 (0.8)
Mycoses co-infection	
Cutaneous mycosis	5 (3.8)

*HIV* Human immunodeficiency virus, *HCV* Hepatitis C virus, *HBV* Hepatitis B virus

* Knott technique and/or skin nips ** coproculture, ***eggs in stool or urine

### Treatment

**Fig. 1:**
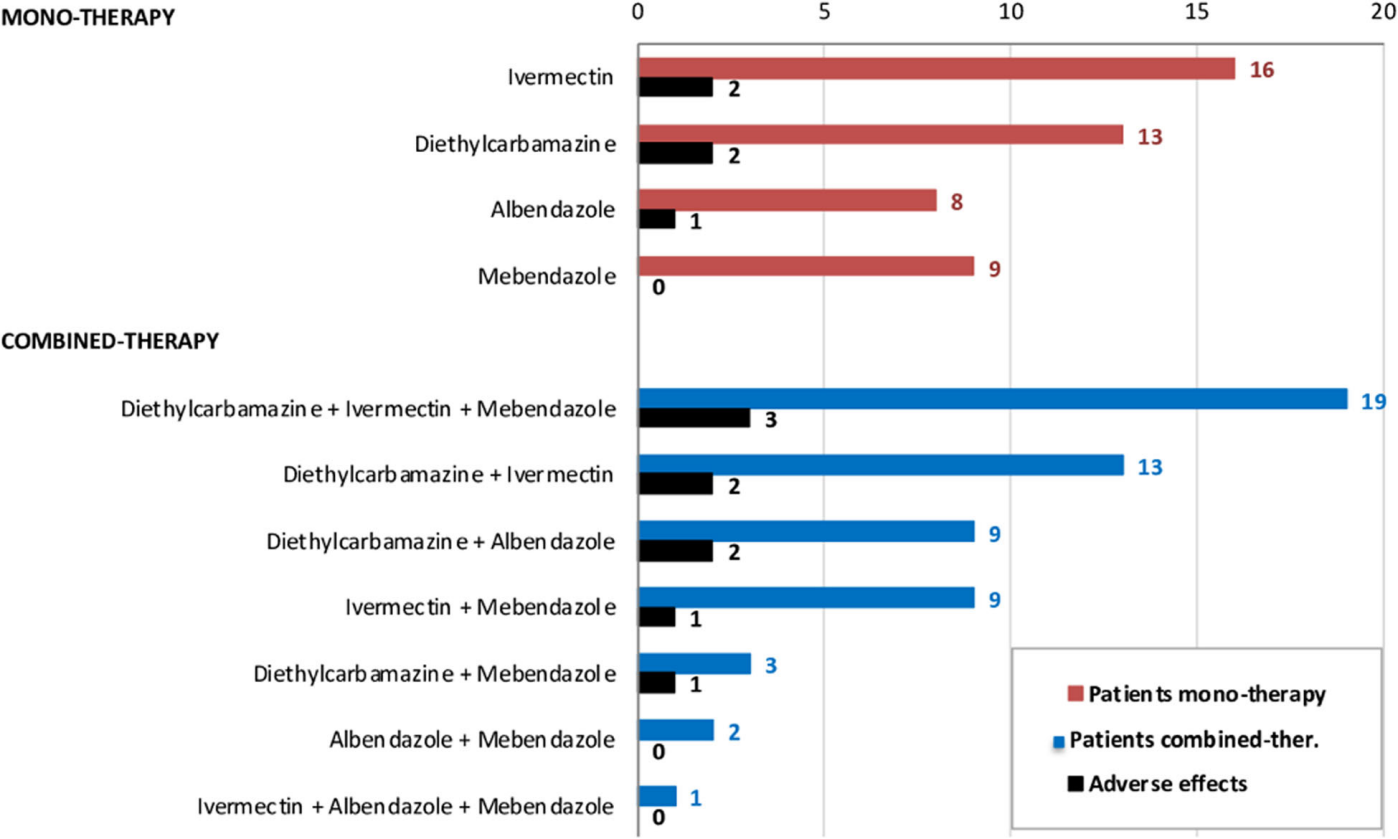
Therapies applied to patients with loiasis in this cohort

In total, 102 patients (77.9%) were treated (Table 4). There were no follow-up data for 29 immigrants (22.1%). There was no information after treatment for 44 patients, and 1 patient failed treatment (albendazole+steroid). Of these 102 patients treated, 46 (45.1%) used only one drug, and 56 (54.9%) used combined therapy: DEC-ivermectin-mebendazole, ivermectin-albendazole-mebendazole, DEC-ivermectin, DEC-albendazole, DEC-mebendazole, ivermectin-mebendazole, or albendazole-mebendazole, as shown in Fig. 1**.** Combined therapy had higher cure rates than mono-therapy: DEC with other antihelminth(s) vs DEC alone (93.2% vs 69.2%, *OR* = 6.0, 95% *CI*: 1.1–32.0, *P* = 0.021); ivermectin with other antihelminth(s) vs ivermectin alone (71.4% vs 0%, *OR* = 3.5, 95% *CI*: 2.2–5.6, *P* <  0.001). In contrast, in terms of side effects, no statistically significant differences were observed between mono-therapy and combined therapy (*P* > 0.05). Corticosteroid therapy was given concurrently with the anti-filarial drug in 44 (33.6%) cases, and an antihistaminic drug was given with the anti-filarial drug in 53 (40.5%) cases.

**Table 4:** Treatment and evolution “cure” in patients with loiasis: mono-therapy vs combined-therapy

	*N* (%) (*N* = 102)	Cure		Adverse effects		Steroids *n* (%) (*N* = 44)	Anti-histaminic *n* (%) (*N* = 53)
		*n* (%) (*N* = 57)	*P*-value* OR (95% *CI*)	*n* (%) (*N* = 14)	*P*-value		
Ivermectin	**58 (56.9)**	**30**		**8**		**19**	**37**
Ivermectin alone	16 (15.7)	0	< 0.001* 3.5 (2.2–5.6)	2/16 (12.5)	0.860	4/16 (25.0)	7/16 (43.8)
Ivermectin with other antihelminth(s)^a^	42 (41.2)	30/42 (71.4)		6/42 (14.3)		15/42 (35.7)	30/42 (71.4)
Diethylcarbamazcine	**57 (55.9)**	**50**		**10**		**32**	**40**
Diethylcarbamazcine alone	13 (12.7)	9/13 (69.2)	0.021* 6.0 (1.1–32.0)	2/13 (15.4)	0.816	9/13 (69.2)	7/13 (53.8)
Diethylcarbamazcine with other antihelminth(s)^a^	44 (43.1)	41/44 (93.2)		8/44 (18.2)		23/44 (52.3)	33/44 (75.0)
Albendazole	**20 (19.6)**	**13**		**3**			**9**
Albendazole	8 (7.8)	4/8 (50.0)	0.251 3.0 (0.4–20.1)	1/8 (12.5)	0.798	5/8 (62.5)	3/8 (37.5)
Albendazole with other antihelminth(s)^a^	12 (11.8)	9/12 (75.0)		2/12 (16.7)		10/12 (83.3)	6/12 (50.0)
Mebendazole	**43 (42.1)**			**5**		**10**	**21**
Mebendazole alone	9 (8.8)	2/9 (22.2)	0.022* 6.4 (1.1–35.9)	0	0.221	0	0
Mebendazole with other antihelminth(s)^a^	34 (33.3)	22/34 (64.7)		5/34 (14.7)		10/34 (29.4)	21/34 (61.8)

*Statistical significance level of 5% (*P* < 0.05)

^a^Combined therapy: Diethylcarbamazcine-Ivermectin-Mebendazole, Ivermectin-Albendazole-Mebendazole, Diethylcarbamazcine-Ivermectin, Diethylcarbamazcine-Albendazole, Diethylcarbamazcine-Mebendazole, Ivermectin-Mebendazole, or Albendazole-Mebendazole

*CI*: Confidential interval; *OR*: Odds ratio

**Fig. 2:**
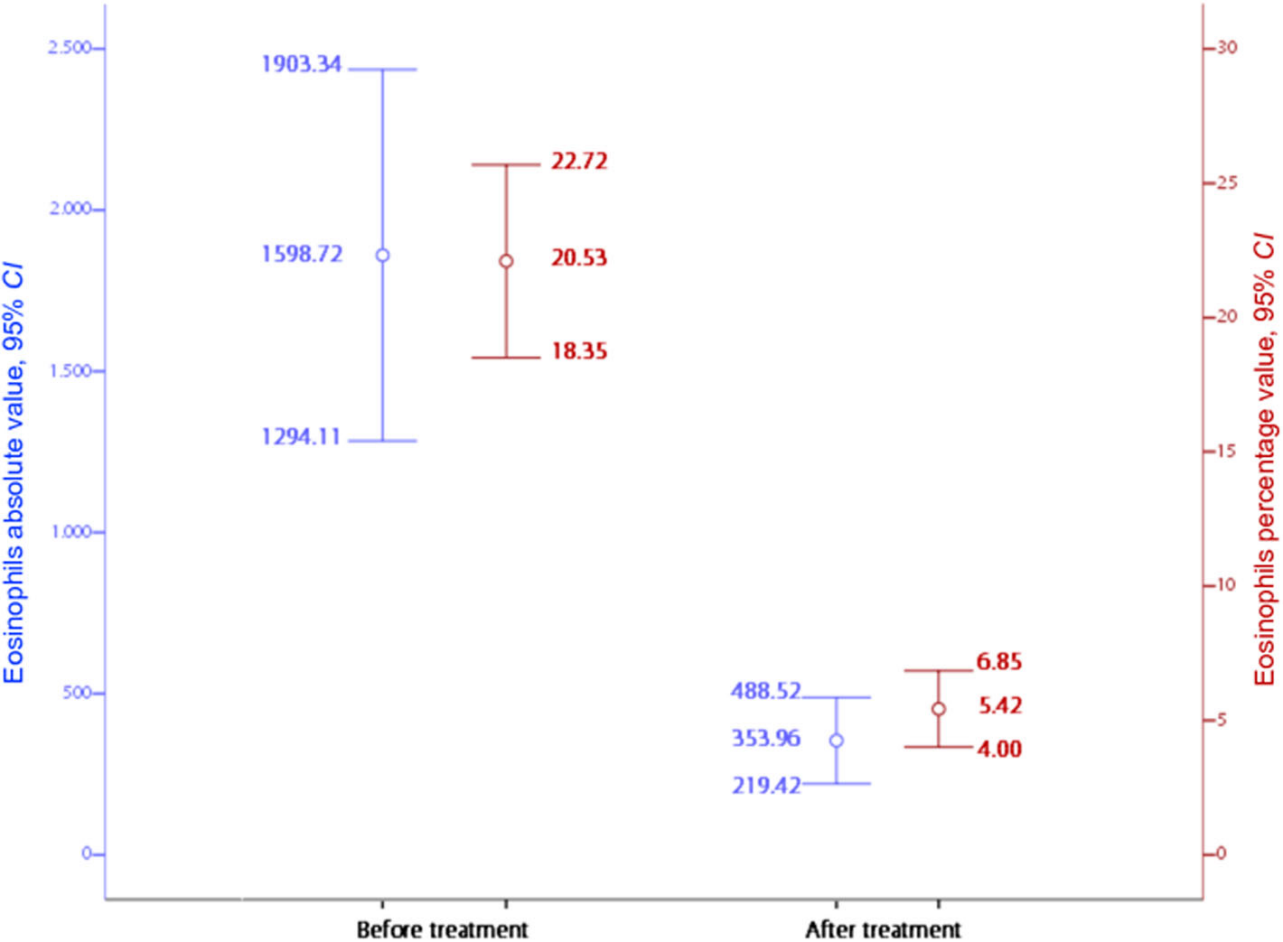
The evolution of absolute and relative eosinophilia (mean, 95% *CI*) after treatment. *CI*: Confidential interval

Figure 2 shows the evolution of absolute and relative eosinophilia after treatment. Adverse reactions were observed in 14 (10.7%) migrants including pruritus (5), Calabar swelling (5), fever and headaches (2), meningitis (1) and hepatotoxicity (1).

## Discussion

Our work has the peculiarity that it is a single-center study focused on migrants. The patients presented early clinical manifestations at the time of assessment, similar to those described in other studies [4, 9, 11–13]. The clinical manifestations described in our work are similar to other studies. The high level of visceral complications and atypical features (nephropathy, rheumatism, pleural effusion) of imported loiasis reported in a recent review by Antinori [13] results from the bias of the literature review, which over-represents unusual cases relative to many unpublished noncomplex cases [9].

Our data show eosinophilia and IgE results that are similar to the study by Gobbi et al. [4]. We do not have the microfilaremia data necessary for a comparison with other studies [4].

Considering the sensitivity of the ELISA assays in our study and despite possible cross-reaction with other filarial infections and strongyloidiasis [9], PCR assays could be helpful for the diagnosis of imported loiasis, but it is not usually available [14, 15].

The diagnosis of imported loiasis is not always easy due to multiple reasons: lack of medical knowledge about this neglected tropical disease, length of the prepatent period, frequency of asymptomatic carriage, possible normal eosinophil count, need for blood puncture around midday for a parasitological diagnosis, and low sensitivity of microscopic detection (absent, transient or low parasitemia, poor experience of thick film). Thus, physicians have to carefully investigate such patients, including for a travel history to sub-Saharan Africa over several years and possible exposure to *Chrysops* bites in cases of unexplained migratory edema.

The first-line drug for the treatment of loiasis is DEC, while in the absence of this drug and in the case of low microfilaremia, albendazole plus ivermectin could be a useful and effective treatment [16]. Our patients used multiple drugs and combinations: DEC, ivermectin and albendazole, alone or successively. DEC is a piperazine derivative that acts against microfilariae and adult worms. Nevertheless, ivermectin has only anti-microfilaricidal activity, and albendazole has only anti-macrofilaricidal activity. These are currently employed for the treatment of loiasis, although several limitations have been described. Adverse events can restrict their use, and they can provoke severe inflammatory responses in patients with onchocerciasis coinfections [13, 17]. The different treatments were usually selected without any specific criteria. It is challenging to propose an evidence-based treatment for imported loiasis considering the limitations of retrospective surveys. We believe that the management of this infection will remain based on personal experience and expert opinion because randomized controlled trials are scarce [4].

There is no consensus about a definition of “cure” in loiasis, although missing clinical signs and circulating microfilaria may indicate that the infection was cured. However, the time between medical treatment and disease control has not been established. Microfilaremia levels can be very low, and blood eosinophilia cannot predict adult worm death. To date, the level of anti-filarial antibodies or the microfilaremia levels determined by qPCR remain speculative in determining if the disease has been cured [9].

## Conclusions

A large case series of imported loiasis in Europe was described. Our patients presented early clinical manifestations and few atypical features. Thus, physicians should systematically consider loiasis in migrants with a typical presentation. However, considering that 72.5% of the patients had only positive microfilaremia without any symptoms, we suggest searching for microfilaremia in every migrant coming from endemic countries for loiasis presenting with eosinophilia. More studies are needed to define the optimal treatment and follow-up.

## Supplementary information

**Additional file 1.** Editing Certificate.

## Data Availability

The dataset supporting the conclusions of this article is included within the article and its Additional file 1.

## Abbreviations

DEC: Diethylcarbamazine
HIV: Human immunodeficiency virus
IQR: interquartile range
*OR*: Odds ratio
*SD*: Standard deviation
