# Supplementary material for: Loiasis in sub-Saharan migrants living in Spain with emphasis of cases from Equatorial Guinea
Source: Infect Dis Poverty. 2020 Feb 7;9:16. doi: 10.1186/s40249-020-0627-4 (PMC7006425; doi:10.1186/s40249-020-0627-4)
Supplement: Supplementary file 1 — Additional file 1. Editing Certificate. [file 40249_2020_627_MOESM1_ESM.pdf]

Editing Certificate

This document certifies that the manuscript

Loiasis in Sub-Saharan migrants living in Spain with emphasis of cases from Equatorial Guinea

prepared by the authors

Sabino Puente (retired)a, German Ramírez-Olivenciab, Mar Lagoa, Mercedes Subiratsc, Francisco Brud , Eugenio Pérez-Blazqueze, Marta...

was edited for proper English language, grammar, punctuation, spelling, and overall style by one or more of the highly qualified native English speaking editors at AJE.

This certificate was issued on **December 18, 2019** and may be verified on the AJE website using the verification code **B47F-8017-713D-6E4A-C3EE**.

Neither the research content nor the authors' intentions were altered in any way during the editing process. Documents receiving this certification should be English-ready for publication; however, the author has the ability to accept or reject our suggestions and changes. To verify the final AJE edited version, please visit our verification page at aje.com/certificate. If you have any questions or concerns about this edited document, please contact AJE at support@aje.com.

AJE provides a range of editing, translation, and manuscript services for researchers and publishers around the world. For more information about our company, services, and partner discounts, please visit aje.com.
